# Equivalent Heat Treatments and Mechanical Properties in Cold-Rolled TiNiFe Shape-Memory Alloys

**DOI:** 10.3390/ma16237395

**Published:** 2023-11-28

**Authors:** Shuwei Liu, Songxiao Hui, Yanfeng Li, Xiaoyun Song, Yang Yu, Wenjun Ye

**Affiliations:** 1 State Key Laboratory of Nonferrous Metals and Processes, China GRINM Group Co., Ltd., Beijing 100088, China; rotund_jere@foxmail.com (S.L.); songxiaoyun@grinm.com (X.S.); yuyang@grinm.com (Y.Y.); wenjun_ye@sina.com (W.Y.); 2 GRIMAT Engineering Institute Co., Ltd., Beijing 101407, China; 3 General Research Institute for Nonferrous Metals, Beijing 100088, China; 4 GRINM (Guangdong) Institute for Advanced Materials and Technology, Foshan 528051, China

**Keywords:** TiNiFe alloy, shape-memory alloy, heat treatment, Avrami model, recrystallization

## Abstract

Heat treatments after cold rolling for TiNiFe shape-memory alloys have been compared. After EBSD analysis and as calculated by the Avrami model and Arrhenius equation, the relationship between the heat-treatment temperature and manufacturing time of TiNiFe alloys is established. Through calculation, it can be found that TiNiFe alloys can obtain similar microstructures under the annealing processes of 823 K for 776 min, 827 K for 37 min, and 923 K for 12.5 min. And the recrystallization fractions are all around 50%. Nevertheless, the tensile properties and recovery stress of the alloys show almost similar values. And based on the feasibility of the annealing process, it is believed that annealing at 873 K for 37 min is the optimal choice to obtain a recrystallization fraction *φ_R_* = 50%.

## 1. Introduction

TiNi shape-memory alloys (SMAs), as important functional materials, are widely used for actuators, coupling joints, aircraft structures, orthodontic appliances, sensors and so on, because of their excellent shape-memory effect (SME) and superelasticity (SE) [1,2,3]. Both SME and SE are based on the martensitic transformation. When the ambient temperature is below the martensitic transformation start temperature (M_s_), the alloy will show SME, while when the ambient temperature is above its austenitic transformation finish temperature (A_f_) and under proper external stress, martensitic transformation will occur, the product of which is named stress-induced martensite (SIM), and the SMA will present SE [4,5,6,7]. Most studies have focused on TiNi binary alloys, while the addition of the Fe element can replace Ni atoms in TiNi unit cells and form an intermetallic compound, leading to lattice distortion and atomic relaxation [8,9]. TiNiFe alloys also have good SME and mechanical properties, besides an extremely low martensitic-transformation temperature [10,11]. There are significant differences in the mechanical properties of TiNiFe alloys at different deformation temperatures and TiNiFe alloys will show a characteristic of multiple steps in phase transformation [12,13]. In addition, the heat-treatment temperature has an effect on the phase-transformation temperature of the TiNiFe alloy and its texture [14]. Cold working can introduce a large number of defects to improve both the strength of the TiNiFe alloy at room temperature and the SME at low temperature [15,16,17,18]. However, the cold-working process will also lead to a sharp decrease in the plasticity of the alloy [7,19]. TiNiFe SMA is a kind of alloy which cannot be strengthened by solution strengthening. Due to work hardening, the TiNiFe alloy is difficult to work cold [16]. Only an appropriate annealing process can make the alloy have excellent plasticity while maintaining a high SME and good mechanical properties [8]. The grain morphology and orientation of the TiNiFe alloy after cold working change dramatically. At the same time, there is no dynamic recovery (DRV) or dynamic recrystallization (DRX) in the cold-working process, and there are defects such as high-density dislocations in the alloy after cold working, which will cause a failure in the specific process used [19]. Therefore, annealing after cold working is particularly important. It was reported that annealing at different times and temperatures could lead to ‘equivalent’ treatments, which would present the alloy as having similar grain morphology and mechanical properties under different annealing processes [20].

TiNiFe alloys annealed after cold working will undergo three stages: recovery, recrystallization and grain growth [21]. And the annealing process can be related to the Arrhenius law and the recrystallization fraction can be related to the Avrami equation. Therefore, an annealing treatment can be obtained with different couples of time–temperature annealing processes through the above two laws.

In this paper, three ranges of different annealing processes have been performed and the mechanical properties, grain morphology and the recrystallization fractions have been compared. From the experimental results, the relationship between the annealing process and recrystallization have been established. And this paper will provide guidance for the control of the alloy recrystallization process by heat treatment.

## 2. Materials and Methods

TiNiFe alloy ingot was prepared by vacuum induction melting, and the 150 mm × 30 mm × 1.1 mm sheet was obtained after cold rolling, and the total reduction of TiNiFe alloys was 27%. The chemical composition of the material is given in Table 1.

To study the effect of annealing process on the microstructures and properties of TiNiFe alloy, the cold-rolled TiNiFe alloy sheets were held at different times *t* and for three temperatures *T*. And they were divided into three different ranges, range I for low annealing temperature, range II for medium annealing temperature and range III for high annealing temperature. Details of these ranges are shown in Table 2.

For microstructure observations, the TiNiFe sheet was wire cut into 5 mm (along the rolling direction) × 4 mm specimens, which were sandpapered and polished by Struers OP-S to mirror-like metallic luster. The microstructure observation and the Electron Backscattered Diffraction (EBSD) were carried out by JEOL JSM-7900F with EBSD probe. And EBSD data was analyzed by TSL-OIM software v7.3.1.

**Figure 1:**
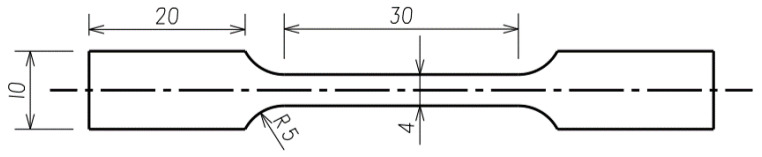
Schematic diagram of tensile samples (unit: mm).

MTS E45.105 electronic testing machine (MTS, Eden Prairie, MN, USA) was used for mechanical properties tests. The tensile test samples were prepared by wire cutting from the cold-rolled alloy sheet, the tensile properties at room temperatures were tested with an initial strain rate of 5 × 10^−4^ s^−1^. As shown in Figure 1, the length of gauge section was 30 mm, and the width was 4 mm. An environmental chamber was equipped for recovery tests; the environmental chamber could provide a temperature condition within the range of 87 K to 773 K. The liquid nitrogen and heating wire was used to adjust the temperature in the chamber. The TiNiFe alloy tensile samples were firstly pre-strained by 8% at 87 K with a quasi-static strain rate of 5 × 10^−4^ s^−1^, after that they were unloaded to free and maintain the strain at this time, and then heated at the rate of 10 K/min for constrained recovery to determine the recovery stress.

## 3. Recrystallization Model Results

Recrystallization kinetics depend on the nucleation rate N and growth rate G. Meanwhile, static recrystallization is actually related to both temperature T and annealing time *t.* Through the Johnson–Mehl formula [22]:

(1) $$\varphi_{R}=1\;-\;\exp\left(\left(-\pi NG^{3}t^{4}\right)/3\right)$$

the recrystallization fraction *φ_R_* after t time at temperature T can be found. But the nucleation rate N is not invariable. Therefore, the Johnson–Mehl formula needs to be modified.

The kinetics of static recrystallization are usually well described by the Avrami model which is derived from the Johnson–Mehl formula [22,23]:

(2) $$\varphi_{R}=1\;-\;\exp\left(-Bt^{K}\right)$$

where *t* is the annealing time at a constant temperature T, and K and B are constants. Taking a logarithm on both sides, we obtain:

(3) $$\mathrm{lg}\ln\left((1/\left(1\;-\varphi_{R}\right)\right)=lgB+Klgt$$

where the slope is K, and the intercept is lgB, by plotting the $\mathrm{lg}\ln\left(1/\left(1\;-\varphi_{R}\right)\right)\;-lgt$ figure.

In addition, the effect of isothermal temperature on the recrystallization rate υ can be expressed by the Arrhenius equation and Avrami model [23,24,25]:

(4) υ =Aexp(−Q/(RT))

and the recrystallization rate υ is inversely proportional to the time *t* required to produce a certain volume fraction *φ_R_*, so:

(5) $$1/t\;=A^{\prime }exp(-Q/(\mathrm{RT}))$$

where A′ is constant, Q is the activation energy of recrystallization, R is the gas constant, and T is the thermodynamic temperature. Taking a logarithm on both sides of the above Formula (5) we obtain:

(6) $$1/T=\;2.3R/Q\times \mathrm{lg}A^{\prime }+2.3R/Q\;\times \;lgt$$

and the recrystallization fraction *φ_R_* = 50% is often used for plotting the figure, where the slope is 2.3R/Q. With this method, the activation energy of recrystallization Q is a constant value. All the parameters can be determined to obtain the following formula:

(7) $$t_{1}/t_{2}=exp\left(-\left(Q/R\right)\left(1/T_{2}\;-1/T_{1}\right)\right)$$

and this formula can be used to calculate the time required to obtain the same recrystallization fraction at different annealing temperatures.

## 4. Results and Discussion

### 4.1. Analysis of the Recrystallization Process

**Figure 2:**
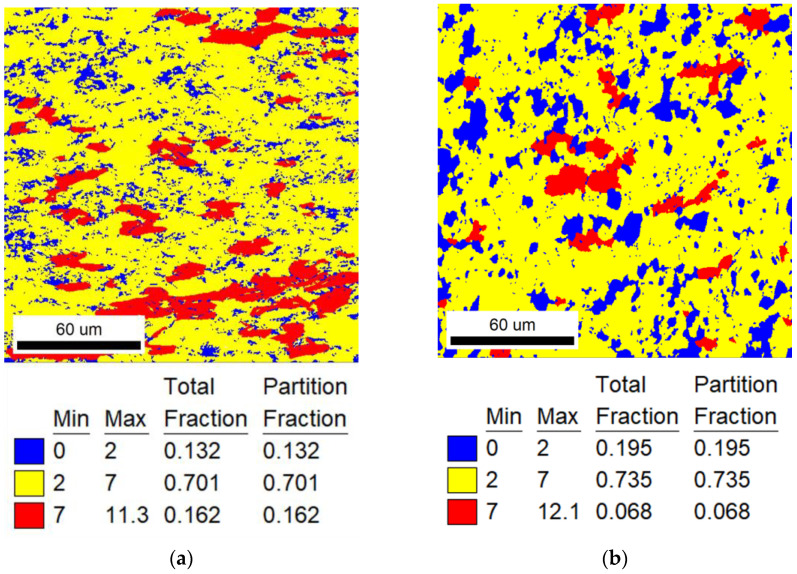

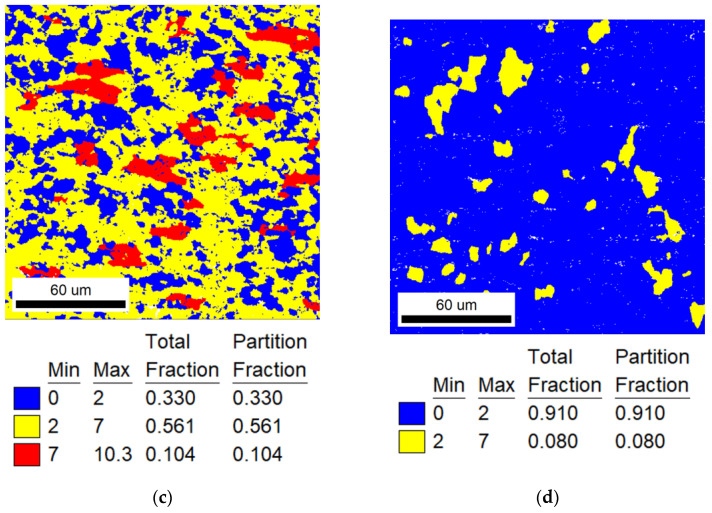
EBSD-GOS maps of TiNiFe alloy used for Avrami model and Arrhenius equation which were annealed at 823 K for different times. Annealed at 823 K for (**a**) 300 min; (**b**) 360 min; (**c**) 720 min; (**d**) 1440 min.

**Figure 3:**
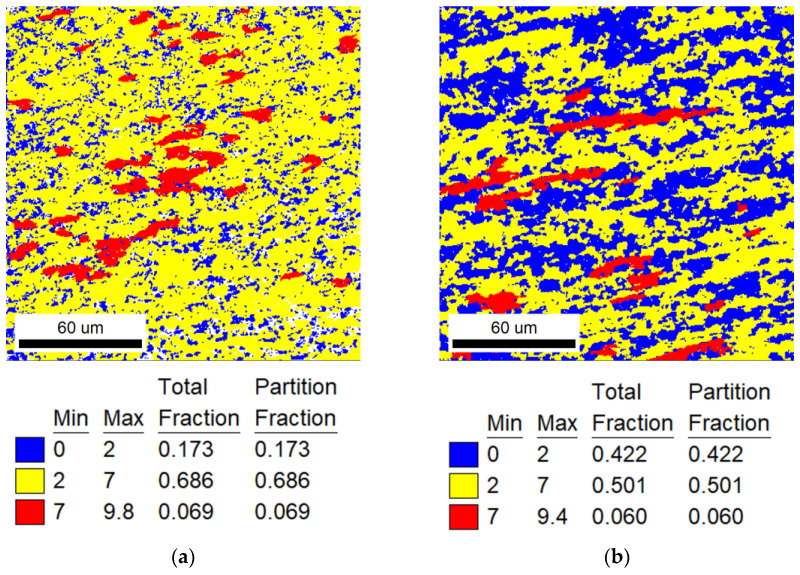

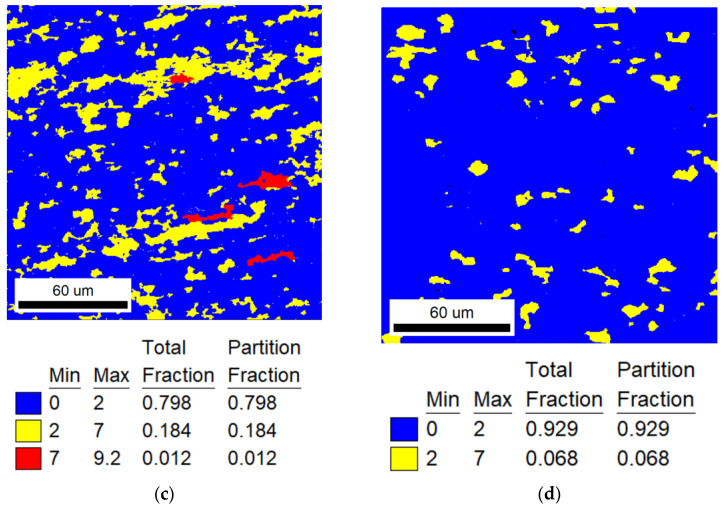
EBSD-GOS maps of TiNiFe alloy used for Avrami model and Arrhenius equation which were annealed at 873 K for different times. Annealed at 823 K for (**a**) 15 min; (**b**) 30 min; (**c**) 60 min; (**d**) 120 min.

**Figure 4:**
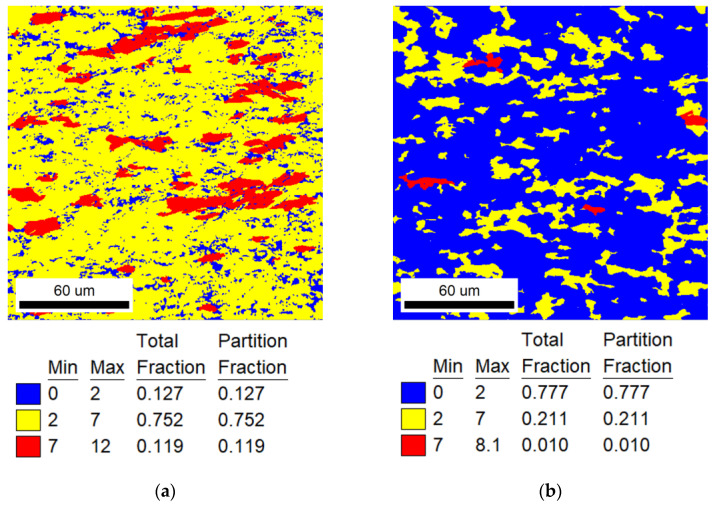

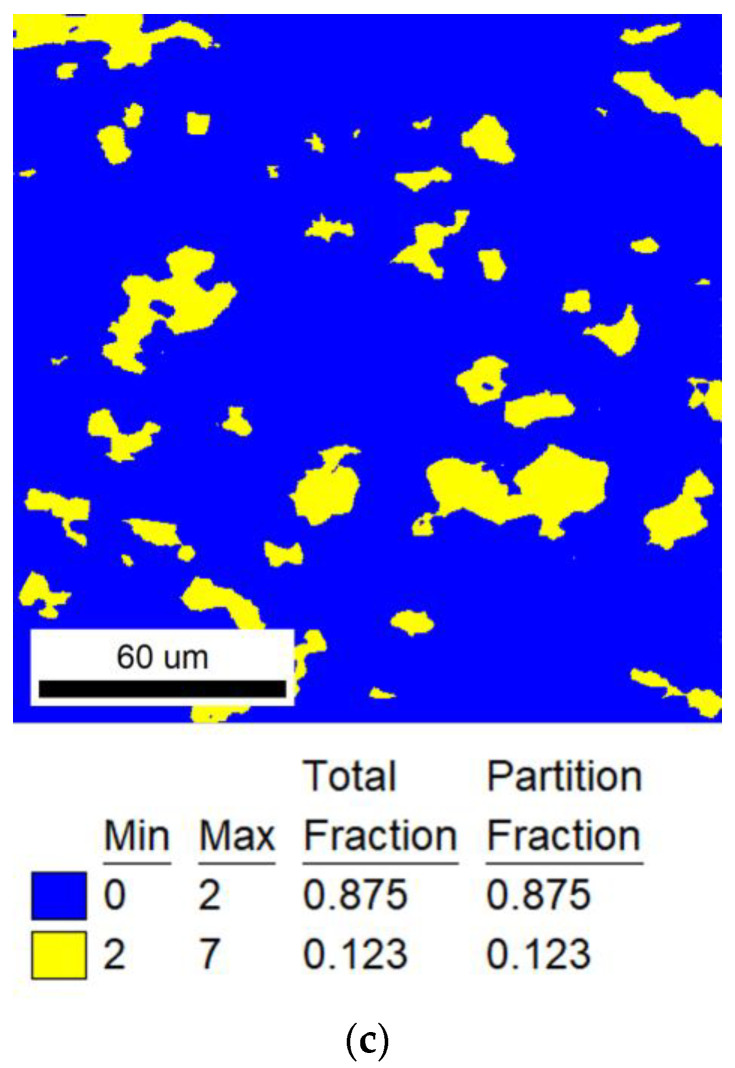
EBSD-GOS maps of TiNiFe alloy used for Avrami model and Arrhenius equation which were annealed at 923 K for different times. Annealed at 923 K for (**a**) 5 min; (**b**) 15 min; (**c**) 30 min.

EBSD analysis was used to calculate TiNiFe alloy recrystallization fractions. EBSD Grain Orientation Spread (GOS) figures are shown from Figure 2, Figure 3 and Figure 4. The recrystallization fraction can be calculated from the EBSD-GOS figures. In our experimental works, by analyzing GOS statistical results and the GOS figures, the recrystallization is considered to occur when the GOS is less than 2, recovery grains are marked for 2 to 7 and deformed grains are marked for 7 to higher. When the annealing temperature is at range I (low annealing temperature for 823 K), the recrystallization process of TiNiFe alloys is extremely slow. It can be clearly seen from Figure 2 that with the increase in annealing time to 300 min, most grains have undergone the recovery process and tended to start the recrystallization process. As the time increases to 1440 min, almost all the grains have been recrystallized. When the annealing temperature reaches range II (medium annealing temperature for 873 K), as shown in Figure 3, the recrystallization inoculation period is short. And severe recrystallization occurs in the alloy after annealing for 30 min. As the time increases to 60 min, only large grains with a small local strain have not been recrystallized. With further annealing to 120 min, the alloy is mainly composed of equiaxed recrystallized grains. As indicated in Figure 4, when the annealing temperature reaches range III (high annealing temperature for 923 K), in particular, recrystallization is completed dramatically in a very short time, and with the increase in annealing time, the grain sizes show a growing trend.

The specific recrystallization fraction statistics of different heat-treatment ranges are shown in Table 3. In addition, it can be found from Figure 2, Figure 3 and Figure 4 that the annealing temperature has a strong effect on the recrystallization behavior of TiNiFe alloys. And there is an important relationship between the recrystallization and the local deformation of grains.

### 4.2. Application of the Recrystallization Model

**Figure 5:**
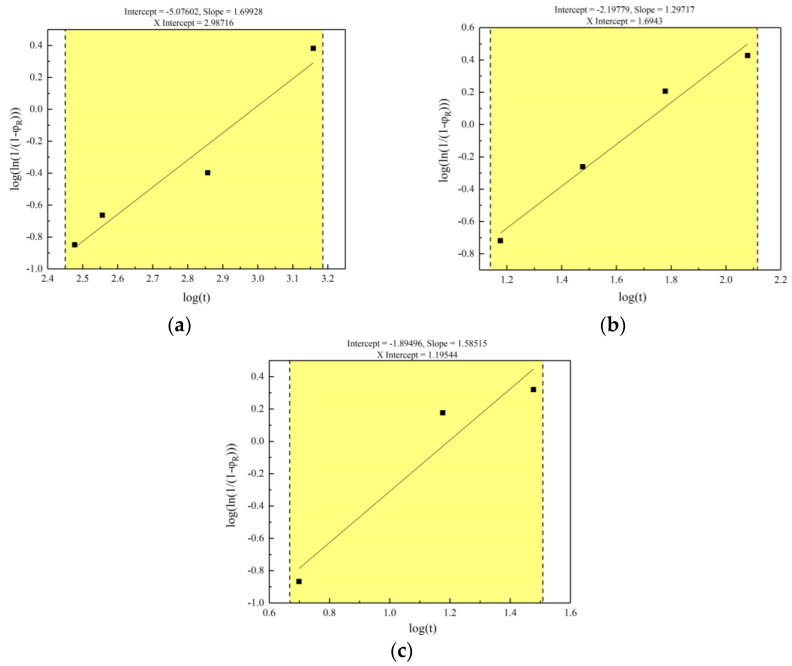
Linear fitting for Avrami models of range I (823 K), range II (873 K) and range III (923 K): (**a**) range I for annealed at 823 K; (**b**) range II for annealed at 873 K; (**c**) range III for annealed at 923 K.

Based on the EBSD−GOS, the recrystallization fractions can be obtained from Table 3. The experimental data of Table 3 are fitted to Equations (2) and (3) which have taken logarithms on both sides. As shown from Figure 5, the agreement between the experimental points and the calculations by Equation (3) is perfect. The relationship between static recrystallization and annealing time at three different heat−treatment temperatures can be known from the fitting results; the Avrami models of three different ranges are shown in Table 4.

**Figure 6:**
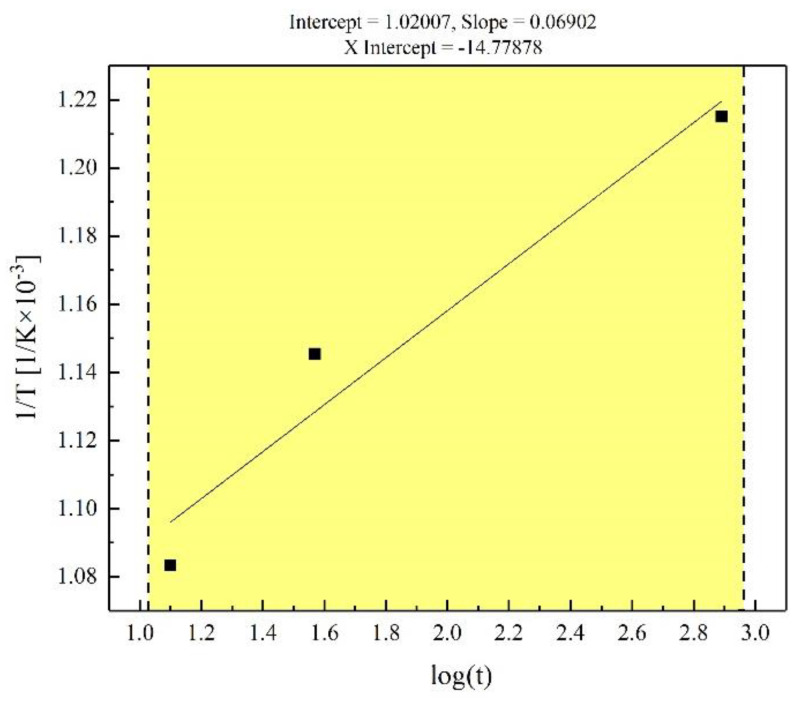
Linear fitting for Arrhenius equation.

From the Avrami models above (Equations (8)–(10)), the time required when the recrystallization fraction *φ_R_* = 50% at different temperatures can be calculated as shown in Table 5. And the annealing time *t* which is calculated from Equations (8)–(10) is brought into Equation (6) and the fitting result is shown in Figure 6. It can be seen from Figure 6 that the straight-line fitting results are highly matched with the previous calculation results.

So far, through EBSD−GOS analysis, Avrami model calculation and Arrhenius equation fitting of three groups of samples, we can obtain the following relationship:

(11) $$1/T=1.02\;\times \;10^{-3}+6.902\;\times \;10^{-5}\;lgt$$

where, at this time, the recrystallization fraction *φ_R_* is 50%, and Q/R can be calculated from Equation (11). Thus, the activation energy of recrystallization Q is as follows: Q = 277.1 kJ. And when we substitute the Q value into Equation (7), the formula for obtaining the same microstructures at different constant heat−treatment temperatures of TiNiFe alloys can be formed like this:

(12) $$t_{1}/t_{2}=exp\left(-33323.7\;\times \left(1/T_{2}-1/T_{1}\right)\right)$$

where *t*_1_ and *t*_2_ are the annealing time and *T*_1_ and *T*_2_ are the annealing temperature. From Equation (12), when the recrystallization condition of the alloy at a certain temperature and time is known, the different heat−treatment methods required to achieve the same microstructure can be obtained by calculation.

### 4.3. Model Validation

**Figure 7:**
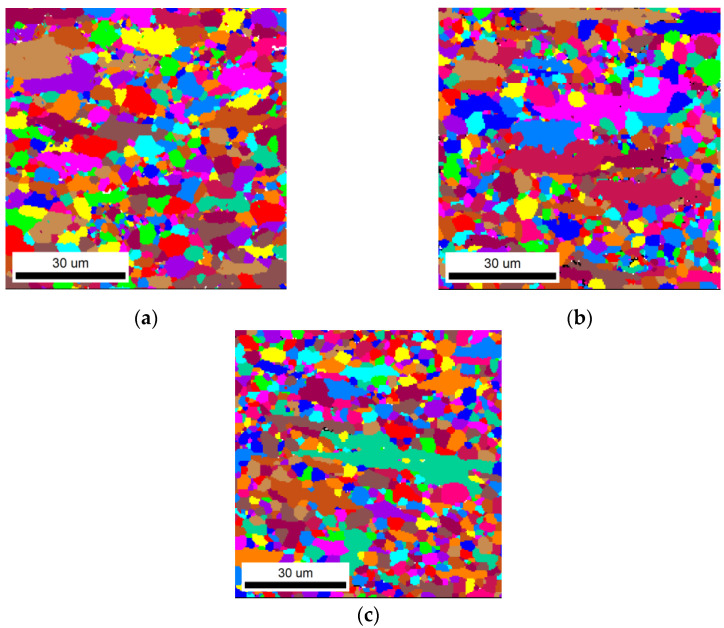
EBSD grain maps of experimental results of recrystallization fractions. The annealing treatments are calculated by Avrami model and Arrhenius equation: (**a**) range I for 823 K, and the annealing time calculation result was 776 min; (**b**) range II for 873 K, and the annealing time calculation result was 37 min; (**c**) range III for 923 K, and the annealing time calculation result was 12.5 min.

**Figure 8:**
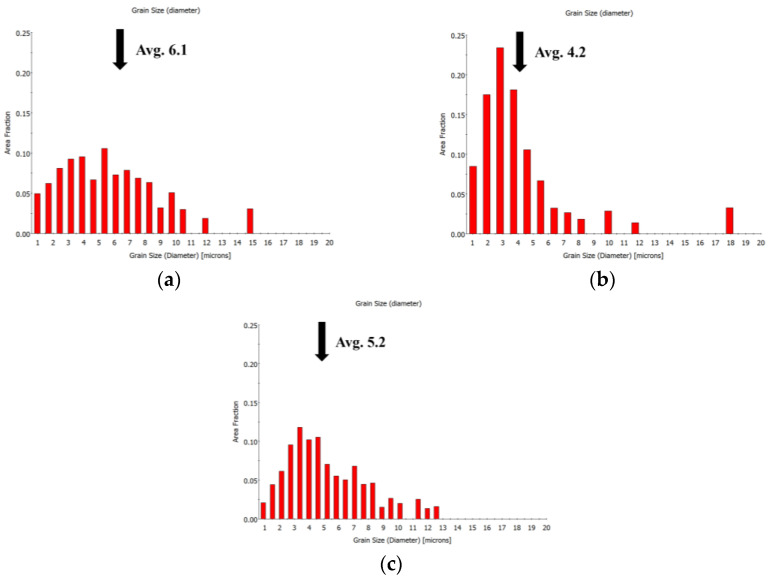
Histograms of the grain sizes and the average grain size (Avg.) of TiNiFe alloy (calculated by EBSD analysis): (**a**) 823 K for 776 min; (**b**) 873 K for 37 min; (**c**) 923 K for 12.5 min.

To verify the accuracy of the calculation results, samples of the calculated recrystallization fraction *φ_R_* = 50% were heat treated according to Table 5. It can be found from the EBSD grain maps that, after the calculated annealing treatment, the grains were mainly fine equiaxed grains, accompanied by some elongated deformed grains along the cold−rolling direction, as shown in Figure 7. Average grain sizes among the three calculated heat treatments are as shown in Figure 8. When annealed at 873 K for 37 min, the average grain size of the TiNiFe alloy is finest (the average grain size is 4.2 μm), as shown in Figure 8b. Meanwhile, the average grain size of the TiNiFe alloy annealed at 823 K for 776 min (the average grain size is 6.1 μm) is slightly coarser than the alloy annealed at 923 K for 12.5 min (the average grain size is 5.2 μm), and the total number of grains with a size less than 5 μm in the former is significantly higher than that in the latter.

**Figure 9:**
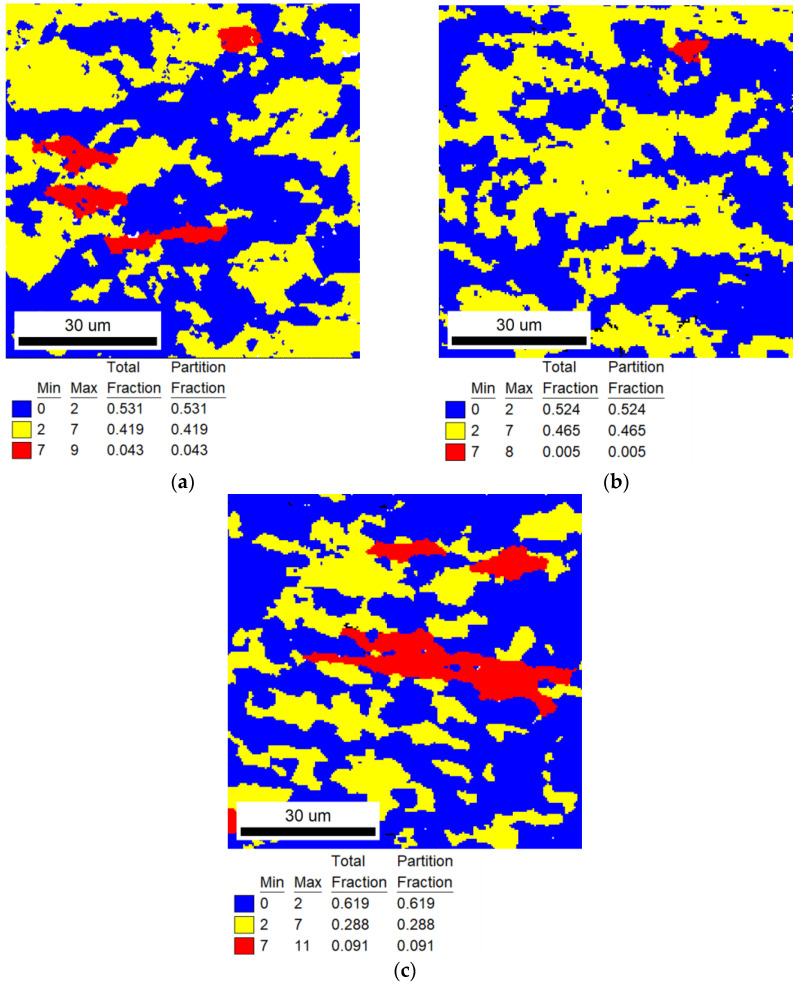
EBSD−GOS maps of experimental results of recrystallization fractions. The annealing treatments are calculated by Avrami model and Arrhenius equation: (**a**) range I for 823 K, and the annealing time calculation result was 776 min; (**b**) range II for 873 K, and the annealing time calculation result was 37 min; (**c**) range III for 923 K, and the annealing time calculation result was 12.5 min.

Based on the EBSD−GOS figures, as shown in Figure 9, the recrystallization fractions can be calculated. Three groups of heat−treated samples at different temperatures can obtain approximately the same structure and recrystallization ratio. The calculated recrystallization fraction *φ_R_* = 50%, and the experimental results are 53.1% at 823 K for 776 min, 52.4% at 873 K for 37 min and 61.9% at 923 K for 12.5 min, as shown in Table 6. The deviations of 823 K and 873 K are less than 5%, and the deviation of 923 K is slightly higher, which is due to the high annealing temperature and the recrystallization process occurring rapidly, resulting in experimental errors. It can be considered that the microstructure (recrystallization fraction) of the alloy meets the calculated formula as shown in Equation (12).

**Figure 10:**
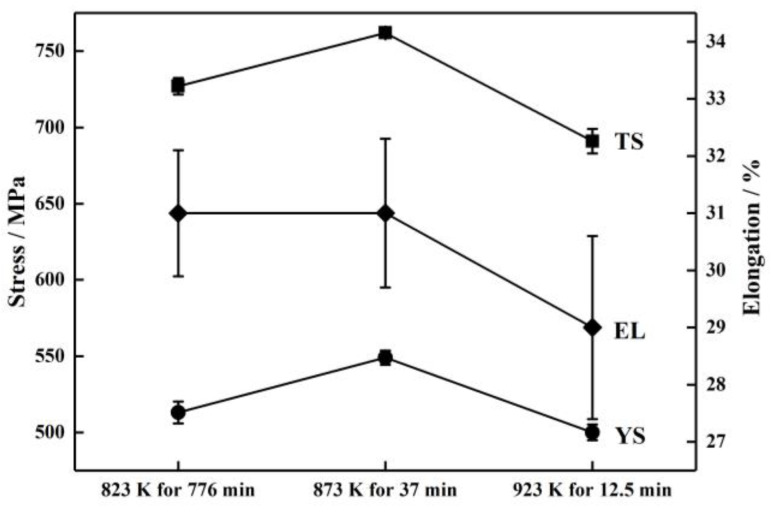
Tensile stress (TS), yield stress (YS) and elongation (EL) of TiNiFe alloy *φ_R_* = 50% with different annealing treatments. The annealing treatments are calculated by Avrami model and Arrhenius equation.

Figure 10 shows the mechanical properties of TiNiFe alloys with different heat treatments as calculated by the Avrami model and Arrhenius equation. From Figure 10, it is illustrated that the tensile strength and yield strength of calculated heat−treatment samples have similar values; the highest tensile strength and yield strength both occur at 873 K for 37 min, and the lowest occur at 923 K for 12.5 min. And Figure 10 also shows the elongation of calculated heat−treatment samples, and all the elongations of heat−treated TiNiFe alloys are close.

It can be seen from Figure 7 and Figure 9 that although the recrystallization fractions of TiNiFe alloys with different annealing treatments are similar, there are still differences in the mechanical properties. This is due to the difference in average grain size among the three calculated heat treatments, as shown in Figure 8. The TiNiFe alloy with the finest grains was annealed at 873 K for 37 min, and the fine-grain strengthening mechanism enhanced the mechanical properties and microhardness. And the reason why alloys annealed at 823 K for 776 min have better mechanical properties and microhardness than alloys annealed at 923 K for 12.5 min is that, although the average grain size of the latter is finer than the former, the former has more ungrown recrystallized grains, which can be seen in Figure 8a which shows a large number of grains with a size less than 5 μm. Therefore, the mechanical properties of alloys annealed at 823 K for 776 min were superior to alloys annealed at 923 K for 12.5 min.

**Figure 11:**
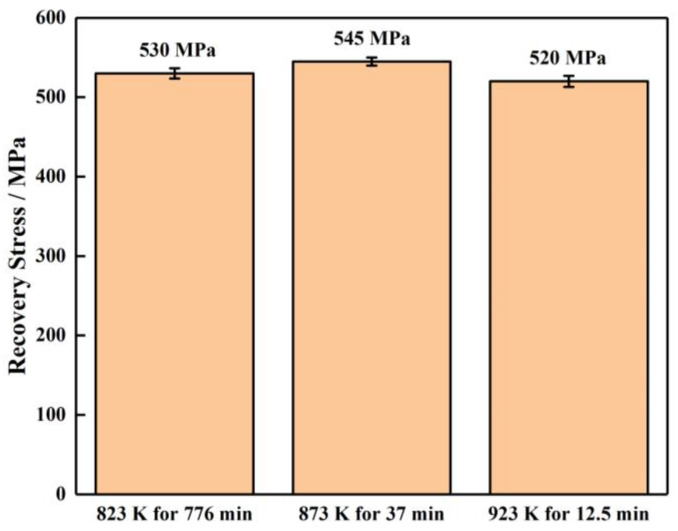
Recovery stress of TiNiFe alloy with different annealing treatments.

Figure 11 shows the recovery stress of the TiNiFe alloy with different heat treatments calculated by the Avrami model and Arrhenius equation. It can be found that the recovery stresses of all the heat treatments as calculated by the Avrami model and Arrhenius equation have similar values. However, as shown in Figure 11, the TiNiFe alloy shows the highest recovery stress after annealing at 873 K for 37 min, and the lowest at 923 K for 12.5 min. This is also related to the grain size shown in Figure 8 above, which indicated that a finer average grain size will result in higher recovery stress in TiNiFe alloys. Therefore, it can be verified that the alloys have similar shape−memory effects under the calculated heat−treatment processes.

The three annealing processes can all obtain similar microstructures and properties, but based on the feasibility of the annealing process, it is believed that annealing at 873 K for 37 min is the optimal annealing process with a recrystallization fraction *φ_R_* = 50%.

## 5. Conclusions

Through this study, it is possible to calculate the heat−treatment process for obtaining similar microstructures under known heat−treatment processes, and to provide guidance for the equivalent heat treatment on the microstructure control of cold−rolled TiNiFe alloys.

1. The formula for obtaining similar microstructures at different constant heat−treatment temperatures of TiNiFe alloys can be formed as Equation (12).
2. We analyzed and established prediction formulas for the microstructure of cold−rolled TiNiFe alloys at commonly used annealing temperatures (823 K, 873 K and 923 K), providing theoretical guidance for calculating the microstructure at different annealing times at commonly used heat−treatment temperatures.
3. After the calculated annealing treatment, cold−rolled TiNiFe alloy sheets have similar microstructures and properties but the sample annealed at 873 K for 37 min has superior microstructures (average grain size is 4.2 μm) and properties (superior TS, YS, EL and recovery stress). Considering the feasibility of the annealing process, annealing at 873 K for 37 min is the optimal annealing process for obtaining a 50% recrystallized structure.

## Figures and Tables

**Table 1:** Chemical compositions of TiNiFe alloys.

Element	Fe	Ni	C	O	Ti
Content(wt.%)	3.27	52.16	0.028	0.031	Bal.

**Table 2:** Heat-treatment processes of TiNiFe alloys.

	Annealing Temperature *T* (K)	Annealing Time *t* (min)			
Range I (Low annealing temperature)	823	300	360	720	1440
Range II (Medium annealing temperature)	873	15	30	60	120
Range III (High annealing temperature)	923	5	15		30

**Table 3:** Recrystallization fraction (GOS < 2) of TiNiFe alloys after different heat-treatment processes.

Annealing Temperature *T* (K)	Annealing Time *t* (min)	Recrystallization Fraction (GOS ≤ 2) *φ_R_*
823 (Range I)	300	13.2%
	360	19.5%
	720	33.0%
	1440	91.0%
873 (Range II)	15	17.3%
	30	42.2%
	60	79.8%
	120	92.9%
923 (Range III)	5	12.7%
	15	77.7%
	30	87.5%

**Table 4:** Avrami model of different ranges of heat−treatment processes.

Annealing Temperature *T* (K)	Avrami Model
823 (range I)	$\begin{array}{c} \mathrm{lg}\ln\left(1/\left(1\;-\varphi_{R}\right)\right)=-5.07602+1.69928\;lgt\;(8) \end{array}$
873 (range II)	$\begin{array}{c} \mathrm{lg}\ln\left(1/\left(1\;-{\;\varphi }_{R}\right)\right)=-2.19779+1.29717\;lgt\;(9) \end{array}$
923 (range III)	$\begin{array}{c} \mathrm{lg}\ln\left(1/\left(1\;-\varphi_{R}\right)\right)=-1.89496+1.58515\;lgt\;(10) \end{array}$

**Table 5:** The time required when the recrystallization fraction *φ_R_* = 50%.

Annealing Temperature *T* (K)	Annealing Time lg*t*	Annealing Time *t* (min)
823 (range I)	2.89	776
873 (range II)	1.57	37
923 (range III)	1.10	12.5

**Table 6:** Experimental results of recrystallization fractions *φ_R_* = 50% and deviations from calculated results.

Annealing Temperature *T* (K)	Annealing Time *t* (min)	Recrystallization Fraction *φ_R_*	Deviation from *φ_R_* = 50%
823 (range I)	776	53.1%	3.1%
873 (range II)	37	52.4%	2.4%
923 (range III)	12.5	61.9%	11.9%

## Data Availability

The data and materials that support the findings of this study are available from the corresponding author upon reasonable request.

## References

[1] Otsuka K., Ren X. (2005). Physical metallurgy of Ti-Ni-based shape memory alloys. Prog. Mater. Sci..

[2] Cai W., Meng X.L., Zhao L.C. (2005). Recent development of Ti-Ni-based shape memory alloys. Curr. Opin. Solid State Mater. Sci..

[3] Otsuka K., Ren X. (1999). Recent Developments in the research of shape memory alloys. Intermetallics.

[4] Mao S.C., Li H.X., Liu Y., Deng Q.S., Wang L.H., Zhang Y.F., Zhang Z., Han X.D. (2013). Stress-induced martensitic transformation in nanometric NiTi shape memory alloy strips: An in situ TEM study of the thickness/size effect. J. Alloys Compd..

[5] Chen C.H., Wu S.K. (2014). Martensitic transformation and pseudoelasticity of aged Ti_50.1_Ni_49.7_Si_0.2_ shape memory ribbon. Mater Sci. Eng. A.

[6] Xu K.F., Luo J., Li C., Shen Y.L., Li C.J., Ma X., Li M.Q. (2022). Mechanisms of stress-induced martensitic transformation and transformation-induced plasticity in NiTi shape memory alloy related to superelastic stability. Scr. Mater..

[7] Yuan X.B., Chen B., Liu F.S., Xu Q., Ma W. (2014). Transformation behaviors and superelasticity of Ti_50_Ni_48_Fe_2_ shape memory alloy subjected to cold-rolling and subsequent annealing. Rare Met..

[8] Lee H.C., Shen J.J., Chang Y.T., Wu C.T., Chen C.H. (2022). Evolutions of superelasticity and elastocaloric effect of Ti_50_Ni_48_Fe_2_ and aged-hardened Ni-rich Ti_49.2_Ni_49.3_Fe_1.5_ shape memory alloys under cyclic compressive deformation. J. Alloys Compd..

[9] Li P.Y., Jia Y.F., Wang Y.S., Li Q., Meng F.Y., He Z.R. (2019). Effect of Fe addition on microstructure and mechanical properties of as-cast Ti_49_Ni_51_ alloy. Materials.

[10] Zhang Y.Q., Jiang S.Y., Zhu X.M., Zhao Y.N., Liang Y.L., Sun D. (2017). Influence of Fe addition on phase transformation behavior of NiTi shape memory alloy. Trans. Nonferrous Met. Soc..

[11] Niu J.G., Geng W.T. (2016). Anti-precursor effect of Fe on martensitic transformation in TiNi alloys. Acta Mater..

[12] Liu X., Li H., Zhang Y.H., Yang Z.W., Gu Q.F., Wang X.H., Zhang Y.F., Yang J.C. (2023). Cryogenic temperature deformation behavior and shape memory mechanism of NiTiFe alloy. Intermetallics.

[13] Tu C.H., Wu S.K., Lin C., Huang B.Y. (2021). A study on two R-phase transformations in intermediate temperature aged Ni-rich TiNiFe-based shape memory alloys. Intermetallics.

[14] Liu X., Li H., Guan H., Yang Z.W., Zhang Y.H., Gu Q.F., Yang J.C. (2022). Degradation of recovery properties after heat treatment in hot-forged NiTiFe alloy. J. Alloys Compd..

[15] Liang Q.L., Zhao S.S., Liang C.X., Zhao T.F., Wang D., Ding X.D., Li S.L., Wang Y.D., Zheng Y.F., Ren X.B. (2022). Strain states and unique properties in cold-rolled TiNi shape memory alloys. Acta Mater..

[16] Ma W., Chen B., Liu F.S., Xu Q. (2013). Phase transformation behaviors and mechanical properties of Ti50Ni49Fe1 alloy with severe plastic deformation. Rare Met..

[17] Liang Y.L., Jiang S.Y., Zhang Y.Q., Hu L., Zhao C.Z. (2018). Microstructure evolution and deformation mechanism of NiTiFe shape memory alloy based on plane strain compression and subsequent annealing. Mater. Chem. Phys..

[18] Jiang S.Y., Yu J.B., Zhang Y.Q., Xing X.D. (2020). Mechanically-induced martensite transformation of NiTiFe shape memory alloy subjected to plane strain compression. Trans. Nonferrous Met. Soc..

[19] Li Y.F., Kang X.Y., Yin X.Q., Xie H.F., Mi X.J. (2014). Microstructure and mechanical properties of cold-rolled Ti50Ni47Fe3 shape memory alloy. Trans. Nonferrous Met. Soc..

[20] Chouf S., Morin M., Belkahla S., Guenin G. (2006). Equivalent thermo-mechanical treatments in an equiatomic Ti-Ni shape memory alloy. Mater. Sci. Eng. A.

[21] Liu S., Li Y., Song X., Yu Y., Ye W., Hui S. (2023). Microstructures and Mechanical Properties of Annealed Ti_50_Ni_47_Fe_3_ Shape Memory Alloy. Crystals.

[22] Johnson W.A., Mehl R.F. (1939). Reaction kinetics in processes of nucleation and growth. Trans. Metall. Soc. AIME.

[23] Avrami M. (1939). Kinetics of phase change. I general theory. J. Chem. Phys..

[24] Avrami M. (1940). Kinetics of phase change. II transformation-time relations for random distribution of nuclei. J. Chem. Phys..

[25] Avrami M. (1941). Phase change, and microstructure kinetics of phase change. III. J. Chem. Phys..

